# ‘*Some of my patients only come to renew their prescriptions. They are not interested in any additional advice or support’.* Physicians’ perceptions on their roles in cardiovascular diseases risk reduction and management in Fiji

**DOI:** 10.1017/S1463423622000779

**Published:** 2023-02-08

**Authors:** Nikansha Kumar, Masoud Mohammadnezhad, Ravneel Narayan

**Affiliations:** 1 School of Public Health and Primary Care, Fiji National University, Suva, Fiji; 2 School of Nursing and Healthcare Leadership, University of Bradford, Bradford, UK; 3 Department of Health Education and Behavioral Sciences, Faculty of Public Health, Mahidol University, Nakhon Pathom, Thailand; 4 Department of Anaesthesia, Colonial War Memorial Hospital, Suva, Fiji

**Keywords:** physicians, primary health care, perceptions, cardiovascular diseases, risk reduction, Fiji

## Abstract

**Background:**

Primary health care (PHC) physicians’ perceptions are vital to understand as they are the first-line health care providers in cardiovascular diseases (CVD) risk assessment and management. This study aims to explore PHC physicians’ perceptions on their roles and their perceptions on management and risk reduction approaches on CVD risk reduction and management in Fiji.

**Methods:**

This is a qualitative study conducted in the Suva Medical area among 7 health centers from 1 August to 31 September, 2021. Purposive sampling was used to recruit physicians who worked in the Suva medical area as PHC physicians with at least 6 months’ experience in the Special Outpatients Department clinics. In-depth interview were conducted using a semi-structured questionnaire over the telephone and recorded on a tablet device application. The interview content was then transcribed, and thematic analysis was done.

**Results:**

This study included 25 PHC physicians. From the thematic analysis, 2 major themes emerged with 6 subthemes. Theme 1 was CVD management skills with 3 subthemes including education, experience and trainings, beliefs and attitudes of physicians, self-confidence and effectiveness in CVD risk reduction and management. Theme 2 was roles and expectations with 3 subthemes including perceptions of effective treatment, perceptions of physicians’ roles and perceptions of patients’ expectations. Physicians generally see their role as central and imperative. They perceive to be important and leading toward combating CVDs.

**Conclusions:**

Physicians’ perceptions on their commitment to prevention and management of CVDs through their skills and knowledge, beliefs and motivation should be acknowledged. It is recommended that the physicians are updated on the current evidence-based medicine. Limitations include results that may not be the reflection of the entire physician and multidisciplinary community and the difficulties in face-to-face interviews due to the coronavirus diseases of 2019 pandemic.

## Introduction

Cardiovascular diseases (CVDs) remain a global priority, and efforts and commitment need to be sustained to achieve the United Nations Global Sustainable Development Goal which aims to reduce premature death by one-third (30%) due to non-communicable diseases (NCDs) (Frieden *et al*., 2020; Roth *et al*., 2020; 2021). The World Health Organization (WHO) defines CVDs as a group of conditions of the heart and blood vessels (Babatunde *et al*., 2020; Cardiovascular diseases, 2021; What is CVD? – World Heart Federation, 2021). These include coronary heart diseases, cerebrovascular diseases, rheumatic heart diseases, peripheral arterial diseases, congenital heart diseases, deep vein thrombosis, aortic aneurysm, cardiomyopathy and pulmonary embolism (World, 2014; Cardiovascular diseases, 2021). The World Heart Federation states that CVDs occur from a combination of factors including socio-economic, cultural, behavioral and environmental (Cardiovascular diseases, 2021; What is CVD? – World Heart Federation, 2021). The modifiable risk factors include tobacco consumption, unhealthy nutrition, harmful consumption of alcohol, physical inactivity or sedentary lifestyle and stress (Definition of cardiovascular disease – NCI Dictionary of Cancer Terms – National Cancer Institute, 2011). The biological risk factors include overweight and obesity, elevated blood pressure (hypertension), diabetes, dyslipidemia and renal diseases (Anderson *et al*., 1991; Mohammadnezhad *et al*., 2016; Ni *et al*., 2019). The non-modifiable risk factors include age, sex, ethnicity and family history (Cardiovascular disease, 2018; Cardiovascular diseases, 2021).

According to WHO, approximately 17.9 million individuals succumbed to CVDs in the year 2019 (Cardiovascular diseases, 2021). This accounted for about 32% of the global mortality out of which 85% of deaths were attributed to stroke and heart attack. WHO also stated that these CVD deaths equate to about 75% of deaths in low- and middle-income countries (LMICs) (Nallamothu, 2017; Cardiovascular diseases, 2021). Pacific Island leaders have declared the region being in NCD ‘crisis’. It is estimated that about 60% of death in the Solomon Islands and 77% in Fiji are due to NCDs. NCDs have led to premature deaths in the Pacific, which is comparable to LMICs (Hou *et al*., 2016).

Fiji has experienced epidemiological transition whereby childhood mortality and infectious diseases are declining and the mortality from CVDs is peaking (Carter *et al*., 2011). Fiji had almost double CVD-related deaths in 2017 and is ranked number 25 in the world with 217.68 per 100,000 of age-adjusted death rates in the general population (Coronary Heart Disease in Fiji, 2021). The NCD status in Fiji after the STEPS survey stated that 19% of the adult age group had hypertension, where 63% were unaware of their disease. Stroke and heart diseases were the leading cause of death in the 40–59 Special Outpatients Department (SOPD) years age group (Fiji-STEPS-Report-2011.pdf, 2021). Although primary care is the major target of CVD clinical recommendations, little is known of how community physicians view their role in management of risk factors and CVD risk reduction and management.

Physicians play a vital role in primary health care (PHC) or public health setting. The Fiji PHC setting is generally managed by the medical officers with a baseline qualification of Bachelor of Medicine and Bachelor of Surgery (MBBS) degree and those who have pursued further studies in Public Health programs. It takes 6 years to obtain the MBBS degree and the Postgraduate (PG) studies continue afterward. Many physicians opt to continue further studies once they are employed in the health system. PG studies are offered at the country’s gigantic university, The Fiji National University, which offers a wide range of programs including public health and primary care. Some physicians get sponsored or opt for education through personal expenses from international universities. These medical officers play a role of general practitioners and seek tertiary care in acute patient management through the major hospitals. Physicians are in the first-line management of diseases in the general and special outpatient’s department. Physicians have trainings on the clinical practice guidelines and continuous medical education during their career beginning from medical school until they join the workforce. Following the internship program, the new doctors serve in the primary care facility and those who prefer enter specialty programs in tertiary care facility while the remaining continue to serve in PHC settings. The integration of clinical and public health approaches to prevention of NCDs particularly CVDs is essential in PHC. The physicians serve through patient education as part of public health interventions. The aim of PHC is to prevent the development of risk factors of CVD in the initial stage (Houston-Miller *et al*., 1997; Fishman *et al*., 2014). An efficient service delivery collaborates members from various disciplines including the nurses, pharmacists, physiotherapists, dieticians’ community health workers, patients, family members and many more. This multidisciplinary care involves communal goals, vibrant roles, trust and efficient communication and determinate development and results (Jennings and Astin, 2017). Multidisciplinary approach to patient care is paramount; however, a physicians’ insight into understanding the preventive and curative strategies stands as the basic foundation to CVD prevention and control. Precise perception of a patient’s risk by doctors is important as this is one of the components that determine health-related behavior (Webster and Heeley, 2010; Brawarsky *et al*., 2018).

Primary care facilitates high-quality critical CVD prevention and management, as this provides prospect to risk assessment and provision of pharmacological and non-pharmacological interventions. However, due to the gaps and challenges in dealing with various patients and inconsistencies in patient enthusiasm and motivation, there is interference in CVD risk assessment and management. Physicians play a role in patient empowerment and at the same time include approaches in view of patients’ risk perception, assertiveness and inspiration to change risky lifestyle behaviors (Bonner *et al*., 2015; Ju *et al*., 2018). Behavior change can also be reinforced and encouraged through counseling services and motivational interviewing techniques by the physicians. This has been shown to be cost-effective and positive action toward prevention and management of CVDs (Lutala & Muula, 2022).

Despite the benefits demonstrated for managing cardiovascular risks, gaps remain in primary care practitioners’ management of risks according to guideline recommendations. Therefore, this study aims to explore PHC physicians’ perceptions on their roles and their perceptions on management approaches on CVD risk reduction and management in Suva, Fiji. This study also aims to make available some new information in CVD risk management in LMICs like Fiji.

## Methodology

### Study design and setting

This study applied a qualitative study design using in-depth interviews via phone calls to explore the primary care physicians’ perceptions on their roles and management approaches on CVD risk reduction. This study was conducted from 1 August to 30 September, 2021, in the Suva Medical area which includes Lami Health Center (HC), Samabula HC, Nuffield HC, Raiwaqa HC, Makoi HC, Valelevu HC and Nakasi HC. These 7 Health Centers provide PHC to about 225,856 people, and approximately 50 doctors are employed across these health centers. These health centers were chosen as they were the most accessible health facilities given the time of the study period and considering the budget of the researcher. The health facilities are also the busiest in the Suva medical area and thus include the experience of physicians with the vast patient exposure and experience with the limited resources.

### Study sample

All doctors working in the health centers in the Suva medical area and participants who could provide in-depth and detailed information about the aim of the investigation were identified and invited to participate in the study. This study included those who worked in Suva medical area as a PHC doctor for at least 6 months and have exposure to conducting SOPD clinic. Doctors who worked in the private sector only, locum doctors working in health centers and doctors who were not willing to participate were excluded. Purposive sampling was used to recruit participants. In this study, 3–4 PHC physicians from each health center, who met the criteria, were chosen for in-depth interviews. Overall, 25 medical officers participated in this study. At 20 participants, data saturation was attained; however, 5 more were included to ensure no important point was missed.

### Data collection tool

A semi-structured open-ended questionnaire was used to guide the interviews. This questionnaire was developed based on literature review and the aim and research questions of the study. The questionnaire included 2 sections: Section 1 includes demographics and general information such as participant’s age, gender and work experience while the second section included 6–7 open-ended questions to understand their perceptions on CVDs risk reduction. Probing questions were added based on the response from the interviewee. Before using this questionnaire, the content validity of the questionnaire was assessed by 3 experts.

### Study procedure

The participants were notified of the study through the Medical Officer-In-Charge (MOIC) at the various health facilities. Invitation letters were sent to all medical officers 1 month prior to the Suva Medical area to participate in the study. Those who met the inclusion criteria and had consented to take part in the study were short-listed for participation in the in-depth interview. A convenient time was arranged with the participants based on their shift of work. Participant information sheet and consent forms were distributed to the participants. Participating officers were interviewed via telephone. The telephone contacts were obtained through the MOIC at the respective health centers. This was in consideration of Fiji’s current battle with a global pandemic, coronavirus diseases of 2019 (COVID-19). The Medical Officers were informed on the interview time and date, and as per their convenience, data were collected. The total time was approximately 30–40 minutes per participant. The interview was recorded on sound recorder app in a tablet device.

**Table 1.** Characteristics of participants based on demographics (n=25)

Characteristics	N (%)
**Age (yrs.)**	
25–34	19 (76)
35–44	4 (16)
45–54	1 (4)
>55yrs	1 (4)
**Gender**	
Male	5 (20)
Female	20 (80)
**Ethnicity**	
I-Taukei	5 (20)
Indo-Fijian	18 (72)
Others	2 (8)
**Years of experience (yrs.)**	
0–5	13 (52)
6–10	7 (28)
11–15	2 (8)
16 and over	3 (12)
**Place of tertiary studies**	
Fiji Islands	22 (88)
Outside Fiji	3 (12)
**Tertiary Qualification**	
Master of Public Health	2 (8)
PG diploma in NCDs	4 (16)
MBBS	19 (76)

* PG – Postgraduate * NCD – non-communicable diseases * MBBS – Bachelor of Medicine and Bachelor of Surgery.

**Table 2.** Response of participants to use of clinical practice guidelines and preference to clinical judgment when managing patients with CVDs

Characteristics	Total participants	Number of participants use clinical guidelines	Number of participants use clinical judgment
**Years of experience (yrs.)**			
0 – 5	13	5	8
6 – 10	7	3	4
11 – 15	2	2	0
16 and over	3	3	0
**Tertiary Qualification**			
Master of Public Health	2	2	0
PG diploma in NCDs	4	4	0
MBBS	19	7	12
**Trainings/CMEs**			
In-house trainings/CMEs	15	6	9
Ministry-based workshops/trainings	10	10	0
**Setting at health facility**		22	3
SOPD*	25 (All participants are based at GOPD as well)		
		5	20
GOPD*			

*SOPD – Special Outpatients Department. These are clinics at public hospitals (non-private) for patients with known medical problems to monitor their condition and ensure ongoing care on a regular basis.

*GOPD – General Outpatients Department. These are clinics conducted at public hospitals (non-private) for the general public needing medical attention for any acute ailments or general medical services.

**Table 3.** Themes and subthemes identified from interviews on perceptions of PHC physicians on their roles in CVD risk reduction and management

Themes	Subthemes
CVD management skills	Education, experience and trainings
	Beliefs and attitudes of physicians
	Self-confidence and effectiveness in CVD risk reduction
Roles and expectations	Perceptions of effective treatment
	Perceptions of physicians’ roles
	Perceptions of patients’ expectations
**Semi-structured Interview Questions:** Section 1: Demographics – Education level, clinical experience, trainings attained, SOPD attachment, self-confidence levels Section 2: Interview Questions • Describe the physicians’ role in CVD risk reduction and management. • Describe the use of clinical practice guidelines and risk assessment tools in their setting. • Describe the challenges faced in managing patients relating to the guidelines and tools and their setting. • Describe the in-cooperation of multidisciplinary approach in CVD risk reduction and management. • Describe the strategies to overcome the challenges above.	

* CVD – cardiovascular diseases.

* SOPD – Special Outpatients Department.

### Data analysis

All interview recordings were transcribed verbatim by the principal researcher and reviewed by the 2 co-researchers. The data were analyzed using manual thematic analysis. Thematic analysis is used in qualitative data analysis. In thematic analysis, the researcher looks at the data closely and finds similar or common themes including topics, meanings and ideas that appear frequently (Caulfield, 2019a).

Thematic analysis was done in steps. After familiarization, the data were systematically coded using repetitive words and phrases. Comparison of data was done to the different categories to determine data coding consistency. An associative sentence from the interview together with an appropriate comment was added to each code. This continued until no new categories emerged. The codes were then organized to develop themes. These themes were constantly checked against each other to ensure they were comprehensible, dependable and unique. These themes were analyzed into conceptual framework to elaborate the fundamental phenomenon. The new data and the existing framework were compared. The final themes were documented in detail.

### Study rigor

For the study process to be trustworthy, the four-dimensional criteria, credibility, dependability, confirmability and transferability, were implemented to assess the quality of the research. The credibility ensured that the study was presented on the elucidation of the experiences of the participants by reviewing the transcripts and identifying the similarities among and within the participants. Transferability ensured that the ability of the findings could be transferred to other setting or research. Dependability ensured that the process was described in sufficient detail so that another researcher could repeat the work. Confirmability is the process to extend the confidence that the results would be confirmed or corroborated by other researchers (Forero *et al*., 2018; Hadfield *et al*., 2018). In this research, all the themes and subthemes together with the study procedure were discussed and reviewed by other co-researchers to increase credibility. The participants’ background and the context were checked through pilot study to increase transferability of the study. The participants were clearly informed on the study procedure both through the information sheet and during consent to ensure dependability. In addition, this research was done with sufficient contribution from the co-researchers for confirmability.

### Ethical consideration

Ethical approval to conduct this study was obtained from Fiji National University’s College Health Research Ethics Committee with the approval ID: 039.21. Approval to conduct the study at the health facilities was obtained from the Ministry of Health and Medical Sciences, mainly from the Divisional Medical Officer. Written informed consent, with rights to withdraw without any consequences, was taken from the participating doctors before collecting data and assurance of confidentiality, and anonymity was provided to them throughout the course of the study and afterward as well.

## Results

### Characteristics of the participants

Twenty-five (25) medical officers participated in this study. Most participants (76%) were at the age category of 25 to 34 years. The gender distribution shows 5 (20%) were male participants and 20 (80%) were female participants. Based on ethnicity, 5 (20%) were I-Taukei (Indigenous Fijians), 18 (72%) were Fijians of Indian descent, and 2 (8%) were from other ethnic background. Majority of participants (52%) had less than 5 years’ experience, while only 12% had more than 16 years’ experience.

Out of the 25, 22 participants were Fiji Island graduates and the other 3 graduated from other countries and were employed here in Fiji with full practicing license. Two physicians had a Master of Public Health degree, 4 had a PG Diploma in NCDs, and the remaining 19 had only the MBBS degrees. In Fiji, MBBS is a six-year course consisting of classroom learning as well as clinical attachments in medical facilities. After completing MBBS, doctors have the option of upgrading their qualifications by pursuing PG studies either in Fiji or abroad (Table 1).

Table 2 shows the frequency of participants based on using clinical practice guidelines and clinical judgment when managing patients with CVDs. Most participants who used clinical judgment had less than 5 years’ experience. Twelve of them who preferred clinical judgment for managing CVDs had MBBS. Among those who used clinical practice, 10 attended the workshops provided by the ministry of health. Most of them (22) used guidelines were working at SOPD.

### Themes and subthemes identified

Two major themes emerged, with 6 subthemes. Theme 1 was CVD management skills, and Theme 2 was roles and expectations (Table 3). The responses from the participants are quoted below with age, ‘FD’ defined as ‘female doctor’ and ‘MD’ defined as ‘male doctor’ as well as their educational status as MBBS for those with undergraduate qualification only and PG for those with PG qualification (PG diploma or Masters).

### Theme 1: CVD management skills

Three subthemes emerged for this theme. These include education, experience and training, beliefs and attitudes of physicians and self-confidence and effectiveness in CVD risk reduction.

#### Education, experience and training

The interview questions were structured to obtain the background of physicians in terms of education, experience and trainings. Based on the fact that the participants were not only locals, their views were also sought regarding the inclusiveness of the Package of Essential Non-communicable Disease Intervention (PEN) model and CVD management guidelines curriculum in their MBBS program.

*‘It would be nice if clinical practice guidelines such as PEN guidelines are introduced to us from medical school. As of now, it’s something we are learning to use on the job’* (28 years old FD, MBBS)

Participants also expressed their experience in dealing with CVD cases during their internship.

*‘Our internship is such that we did not have any exposure to primary care management. Focus is only on curative medicine and not much on preventive medicine… From this year we are having 2-year internship and part of second year of internship incorporate 3 months of Health Center attachment… I guess this will go a long way in building confidence of young graduates before they start working independently as medical officers’ (35 years old FD, PG)*

One participant highlighted the need for medical schools to review their undergraduate curriculum.

‘I believe the curriculum needs to be revised in medical school. And not just medical school, the concept should be well-informed during transition to rural postings. This is something that is lacking in our setting. I, myself, had so much difficulty in associating with the public health approach to CVD prevention and control’ (29 years old FD, MBBS)

Another participant emphasized on the need for continuous medical education.

‘…even if we are not trained, these sessions can be incorporated in our health center-based CME sessions because we know everyone can’t get the proper trainings together. CME is another good way of grasping new concepts, learning new medicine and actually a refresher’. (28 years old FD, MBBS)

Another participant highlighted the importance of having counseling or motivational training during their postings to the PHC settings.

‘I only know about motivational interview through the PEN training. It would be ideal to have those formal sessions as this will allow us to communicate with our patients more effectively…we understand them better when they tell us about their problems instead of us asking where it all lies…but we lack the skill in this technique. Even counselling is an effective way to deal with such problems…’ (36 years old FD, PG)

#### Beliefs and attitudes of physicians

The participants were asked to outline the modifiable and non-modifiable risk factors of CVD. Majority of the physicians were able to correctly list the modifiable and non-modifiable factors for CVDs.

‘So…modifiable risk factors include smoking, obesity, unhealthy alcohol consumption, diet, physical inactivity… On the other hand, non-modifiable factors include sex, age, ethnicity….’ (26 years old FD, MBBS)

Another responded stated:

‘…risk factors are modifiable and non-modifiable…so SNAPs, that is, smoking, poor unhealthy diet, alcohol consumption, physical inactivity, stress and major contributors…there are age, ethnicity, gender, etc…yeah, I think those are the risk factors’. (50 years old FD, PG)

A participant mentioned on the associated physiological factors:

‘The modifiable risk factors such as diet, alcohol consumption, smoking, physical inactivity led to obesity, hypertension and diabetes…also yes, dyslipidemia…these are all causes of CVDs’. (28 years old MD, MBBS)

Another response was:

‘…in our population it’s usually the males more than age of >40, hypertension and diabetes, background history of hypertension and diabetes and smoking history…’ (27 years old FD, MBBS)

The participants were asked to list all the clinical practice guidelines regarding CVD management and how often they use these guidelines in their practice.

‘I am aware of the WHO-PEN, CVD management guidelines and the Fiji Diabetic guideline…I use the guidelines on patient-to-patient basis mostly’. (27 years old FD, MBBS)

Another participant mentioned:

‘I know about cardiovascular guidelines, the PEN guideline…however, I do not use these guidelines in my practice as I do not have any formal training. I prefer to use my own clinical judgment to determine the severity of risk factors’. (32 years old MD, PG)

A participant outlined the Fiji-Cardiovascular Risk Assessment and Management (CRAM) guideline:

‘…and then we have the Fiji-CRAM. This is the one I have heard about from my senior medical officers. I think this is the one that is the combination of all other guidelines in Fiji’. (29 years old FD, PG)

Another participant mentioned the heart score criteria:

‘We were briefly introduced to the heart score during internship during our emergency department rotation’. (27 years old FD, MBBS)

Physicians were also asked in which clinical settings they use those guidelines.

‘So, I only use these guidelines when I am doing SOPD clinics. It becomes difficult to use them in GOPD settings as the utilization is very time-consuming’. (29 years old FD, PG)

The participants were also explored on their instincts of doing the risk assessments on patients on their first encounter, for example, who they would be assessing and who would benefit from these risk assessment and management

‘I will routinely not evaluate patients for CVD risk unless they are presenting with symptoms such as chest pain, headache, weakness, dizziness…I will also investigate further for those who are displaying more than 2 risk factors that I will be able to modify…these are the ones who will benefit more from any intervention…’ (28 years old FD, MBBS)

Another respondent stated:

‘I also believe that if a patient is concerned and is requesting for an evaluation, it is my responsibility to do so’. (29 years old FD, MBBS)

Another participant responded as:

‘I would screen patients with age >40 years especially males, those with significant family history of CVDs, diabetics, heavy smokers, those with sedentary lifestyle, hypertensive etc…Generally those I feel are at high risk’. (29 years old MD, PG)

One participant stated that:

‘…the aim is to reduce the cardiovascular event in the future…we try and address each and every risk factor…that’s where WHO tool comes in…40 plus Indian male with uncontrolled hypertension, uncontrolled diabetes, I would follow them up on a regular basis…for example weekly or two weekly reviews until I have a little bit of control over their pressure or diabetes, then I would refer them to SOPD…’ (27 years old FD, MBBS)

Participants were also asked to provide their opinions on the current available CVD risk assessment tools and guidelines.

‘I guess the tools and guidelines are pretty good. They serve as a guide and makes decision-making easier, especially when it comes to calculating risks…or for cases where you see a patient having ESKD, then you would know which medications to opt for. In fact, they make our work easy’. (26 years old FD, MBBS)

Another participant also stated:

‘…I am glad we have this. This is easy and would just be applicable to patient of any background…be it ethnicity, age, gender etc.…’ (28 years old FD, MBBS)

Some participants said otherwise:

‘…I mean, yes, they are good. But it has more limitations than advantages. You don’t have them available at all times, they require time…you need blood results like cholesterol especially…and I think they don’t match with our setting. We are Fijians, we have a “eat and live” culture. So, I guess we are underestimating ourselves and this would mean nothing…’ (32 years old MD, MBBS)

The Fiji-CRAM knowledge is lacking among most participants.

‘The Fiji-based once I think we need more awareness on them. Now that you have mentioned Fiji-CRAM, I have no idea about it…I just know PEN and the CVD guideline. So, I can’t comment much except that the once I am using are pretty good’. (29 years old FD, PG)

#### Self-confidence and effectiveness in CVD risk reduction

In this study, the participants were gauged on their level of self-confidence in managing a CVD using a numerical rating scale. The rating scale used involved a target from 1 to 10. ‘1’ was allocated as ‘low self-confidence level’, and ‘10’ was rated as ‘high self-confidence level’.

‘…a 7.5. I have been trained…but there hasn’t been any refresher trainings for me in 2021, plus, as it is, I usually am attached to general outpatient; patients to be seen daily instead of SOPD clinics, so at times does become difficult for me to assess and treat patients in GOPD as I do not have the proper assessment tools with me in general outpatient, hence I have to refer to the special outpatient doctors to treat my cardiovascular risk patients’. (28 years old FD, MBBS)

A participant with low SOPD exposure stated:

‘I rate myself 6. This is because I do not routinely see these patients therefore, I don’t think I will be assessing them as good as someone who does SOPD clinics daily’. (32 years old MD, MBBS)

Another participant stated that despite not having any training she still feels confident.

‘I haven’t had any formal trainings apart from the CME sessions at work, however, I rate myself as 8 as I believe I am able to identify the risk factors and manage patients well’. (38 years old FD, MBBS)

The participants’ follow-up question was to identify how they can gauge the outcome of their treatment plans. The responses included the following:

‘…by having regular follow-up, I see the trend in my patients’ blood sugar, blood pressure, cholesterol level, weight, to gauge the effectiveness. I make the patient record books and give to them to bring on every visit to a health facility’. (28 years old FD, MBBS)

Few participants reported use of color coding:

‘I use the color-coding system. I see the difference in their color as per the PEN protocol. So, if the color changes from orange to green or yellow, I see the patient is doing pretty well’. (32 years old MD, MBBS)

### Theme 2: Physicians’ Roles and expectations

Three subthemes emerged for this theme. These include perceptions of effective treatment, perceptions of physicians’ roles and perceptions of patient’s expectations.

#### Perceptions of effective treatment

In order to explore the participants’ acceptance to the guidelines and tools, they were given three clinical case scenarios’ and asked to assess and manage the cases. The interview questions include the application of tools and guidelines to explore the factors contributing to CVD risk reduction and management.

The participants responded as below:

‘I advise the patient what the risk is, what kind of changes do they need to make in their life, and to see if they need any pharmacological interventions or not… and if there are any changes to their medications, I need to advise them’. (38 years old FD, PG)

Another participant stated following of guideline.

‘…this is basically I would say for those with risk less than 10%, I might, might not…like few decisions, but those which are high risk, yes definitely use the protocols that are in place, from my side, I will follow, but then the final decision is made through patient’s consent. If they are willing for those medications that I am going to start on them, they are happy to take it, then I will go ahead. But if these patients are not wanting to be on certain medications for their own reasons, it’s their choice. After counselling, they still don’t want it, then I won’t be forcing the medications on their list’. (35 years old FD, PG)

Participants were asked if our health system was keeping up with a more advanced or a developed health setting where the resources are more readily available. Majority of the response was that our health system is lagging.

‘In my opinion, no! Our health system is not keeping up with these ideal practices …our area population is so large and there are so many patients coming in for SOPD so ideally you are supposed to spend at least 20 minutes or 30 minutes per patient so that you can do an optimal clinic for those patients, so we are unable to that… It terms of follow-up; the follow-up is usually in 5 months or 6 months even for those who are like assessed as having red or brick red, and it should be like every 3 months or every 4 months…that’s the place where we are not ideal’. (36 years old FD, PG)

Another participant stated poor resources limited the application of guidelines:

‘Our guidelines are developed following models from more developed centers, which is good. However, we are not equipped with appropriate resources to put the guidelines in practice. So, I would say that while the guidelines may be keeping up with other parts of the world, practice is certainly far from what is seen in the developed parts of the world’ (32 years old MD, MBBS)

The participants were asked on how they see their management plans for the patients and if they could meet the targets of their protocols.

‘Some patients, they are willing to change their lifestyle… they are willing to listen to us… and at times, some patients it’s very hard to change their mindset and their compliance to medications’. (38 years old FD, PG)

One participant claimed high compliance to management plans:

‘…at least 80% of the patients are compliant. The rest 20% are non-compliant and there are reasons why they are not compliant. The specific reason that I come across is that; one is the age factor, they tend to forget the time the medications is supposed to be taken, the dosage they are supposed to take and then there is a lot of medicine, so they become quite choosy, so that’s poly-pharmacy’. (28 years old FD, MBBS)

Few participants reported to using objective parameters for evaluation:

‘…I gauge my patients in terms of the therapeutic values of risk factors, especially blood pressure readings, glucose readings and lipid profile. I also use the color-coding system for those who do not have complication illness. Sometimes, I notice the color coding is yellow from oranges once all these factors are plotted in graph’. (29 years old FD, PG)

Majority of the participants believed that it is easy to measure therapeutic goals for their CVD patients through follow-up clinics.

‘It’s not difficult to monitor progress of patients as most of the variables such as blood pressure and sugar tests are available all the time… some patients also have home based monitoring. Based on these results we are able to adjust our management plans if indicated’ (32 years old MD, MBBS)

#### Perceptions of physicians’ roles

The participants’ perception of their role in CVD risk management is generally the same.

‘…very important role especially in assessing their risk before any complications come… also managing them before complications arise, putting them on the right medication, at the right times, early intervention…providing primary advice as well before patients have end organ damages’. (37 years old FD, PG)

Another participant stated almost the same comment:

‘I believe it’s the physicians who can make the most difference. They are the guides. Early diagnosis through screening clinics or risk factors can be appreciated by the physicians and early intervention can slow down the process of complications’. (29 years old FD, MBBS)

A few participants highlighted the role of enforcing behavioral changes among the patients. These include the importance of counseling and in cooperating motivational interviews during the clinic.

‘Our clinics are always full…so we do not have enough time and space to talk to all patients regarding their health… counselling will go a long way, but this is usually brief by doctors. Mostly, only dieticians do their part, and the rest is left on the patients to decide. We need to have more time…maybe involve counselors to talk to patients on their risk behaviors, especially smoking and alcohol’. (28 years old FD, MBBS)

Another participant stated that motivational interviewing is the ideal way to deal with CVD risk reduction and management.

‘Motivational interviewing is part of the PEN training…but we cannot include that as part of our clinic time…we see more than 20 patients in a day in clinic…MI takes minimum, maybe, half an hour…it is not realistic in our setting. We can only do a brief of SNAPs counselling within the 10 to 15 minutes we have per patient…that is it’. (32 years old MD, MBBS)

Another participant stated that CVD risk management is not only about physicians.

‘I strongly believe it’s not just physicians. It’s not a one-man army fight. Yes, physicians do their part, but the idea is the multidisciplinary approach. Every health care giver the patient comes through play an important and equal role, from the doctors to nurses to pharmacists to dieticians and physiotherapists’. (32 years old MD, MBBS)

The participants’ general knowledge on the CVD on global and local facts and figures was also explored.

‘I cannot exactly remember the values but definitely NCD is a concern both globally and locally’. (29 years old FD, PG)

Another physician had a similar response:

‘I don’t have the values on top of my head but certainly it’s the leading cause of morbidity and mortality locally and worldwide. I remember sometime back; Fiji was noted to be having highest prevalence of diabetes globally…this is very concerning’. (32 years old MD, MBBS)

Another participant shared her experience with cases that highlight the seriousness of CVDs:

‘…I think Fiji is in the top 5 when it comes to cardiovascular disease and obesity, which is quite alarming for a small country to have such high numbers of cardiovascular disease…I think it’s the biggest killer…the number of young patients coming in…last week I admitted a 30-year-old Indian male with a STEMI…it’s just unbelievable’. (27 years old FD, MBBS)

#### Perceptions of patients’ expectations

Participants reported that they believed patients had high expectations from them.

‘Patient expectations are very high of our health system. They do place a lot of trust in us to be able to help them. There are obviously some who are unappreciative and try to argue on points they had read on Google… My clinical responsibility for each patient remains same and I involve them in formulating their management plans’. (29 years old FD, PG)

Another patient responded that:

‘Most of my patients appear motivated and attend clinics on time. They always have questions about their condition’. (55 years old MD, MBBS)

Some physicians had a different opinion.

‘…some of my patients only come to renew their prescriptions. They are not interested in any additional advice or support. This however doesn’t discourage me from seeing them in a complete manner, that is, assessing them on each visit and managing them as per assessment’. (28 years old FD, MBBS)

Another participant stated that:

‘…patients… they expect us to prescribe them right medications that can magically get rid of all their problems while they are not making any behavioral changes or any lifestyle changes…unfortunately the expectations from patients is that when they come in with uncontrolled hypertension or diabetes that whatever pills we prescribe should get rid of the problem…or if they are having chest pains or they developed a stable or unstable angina, we should be able to get rid of the problem’. (27 years old FD, MBBS)

Ethical issues have also been part of CVD risk management.

‘…we do have ethical dilemmas in the sense that patients wanting to sign out…for example I had a patient with acute pulmonary edema…we stabilized him, and he wanted to sign out, and he did sign out. So, the problem there is we tried our best to explain it to him, but he still wanted to sign out. So, the ethical dilemma there for me was do I respect the patient’s wishes, or do I do what needs to be done to save his life…in the end it becomes very difficult’. (27 years old FD, MBBS)

One participant spoke about justice:

‘…yes, definitely…firstly, our health system is free of cost…so anyone is welcome to seek medical care. It is justified. It is equal’. (55 years old MD, MBBS)

## Discussion

Generally, this study showed that participants who have >20 years of experience still use the guidelines when compared to the younger physicians with fewer years of experience. These less experienced physicians do not prefer to use the guidelines but rely on their clinical judgment to make decisions. This is the unique finding of this study because some studies have stated that there is an inverse relationship in the years of experience and clinical decision-making in accordance to the guidelines (Doroodchi *et al*., 2008; Halabi *et al*., 2020). A vignette survey on CVD risk management in the United States in 2006 found that physicians with ≤10 years of practice were more likely to make clinical decisions in view of the guidelines to manage CVD patients compared to those ≥10 years of practice (Doroodchi *et al*., 2008). Another systematic review showed there is a decreasing clinical performance with an increase in years of experience (Systematic Review: The Relationship between Clinical Experience and Quality of Health Care, 2005; Christian *et al*., 2006).

This study shows that those with PG qualifications tend to adhere to guidelines more than those without any formal PG qualification. Those with background of training through workshops tend to use guidelines more frequently compared to those with no training. A study done in China on the latest knowledge levels on heart failure found that physicians’ with greater qualification had significantly greater awareness of the guidelines on heart failure compared to those with lower qualifications (Gan *et al*., 2017). Similar studies have been done on physicians’ adherence toward guidelines (Berg *et al*., 1997; Cabana et al., 1999; Milchak et al., 2004).

Physicians demonstrated fair understanding of CVD risk factors and were able to appropriately categorize risk factors as either modifiable or non-modifiable. A study among health care workers in South Africa showed poor understanding and knowledge on NCD risk factors (Amadi *et al*., 2018; Onagbiye *et al*., 2020).

Most participants were aware of at least some CVD management guidelines; however, its utility varied among the participants. Some of these guidelines include WHO-PEN, Fiji-PEN and Fiji-CRAM. WHO-PEN means the Package of Essential Non-communicable disease interventions. This framework is for low-resource PHC settings (Gupta *et al*., 2013). The Fiji Ministry of Health adopted the WHO-guided PEN into their CVD guideline, known as the Fiji-CRAM guidelines. A combination of the WHO-PEN guideline, Fiji Diabetes Guideline and Fiji CVD Guideline make up the Fiji-CRAM guideline (Service).

Clinical practice significantly depends on the patient load. Almost all who use the guidelines mentioned that they use it in SOPD clinics only because of time constraints in General Outpatients Department. The findings of this study suggest that physicians who have less patient load are likely to use the guidelines. A survey among the physicians demonstrated that physicians who attended to fewer number of patients with conditions like hypertension and dyslipidemia were more likely to make clinical decisions based on the recommendations of the guidelines (Doroodchi *et al*., 2008; Jasim *et al*., 2017).

The participants were also explored on their instincts of doing the risk assessments for patients on their first encounter, for example, who they would be assessing and who would benefit from these risk assessment and management. Most participants believed that all patients who are symptomatic for CVDs, those requesting to be screened, those who demonstrated high-risk lifestyle behaviors and those with strong family history would be ideal to do risk assessment on and would benefit most. These factors identified by the participants are consistent with recommendations from the WHO-PEN guideline (Gupta *et al*., 2013).

The level of self-confidence in managing CVDs using a numerical rating scale from 1 (‘low self-confidence level’) to 10 (‘high self-confidence level’) was used. The average rating score was 7. Among those who rated themselves higher (score > 7), common reasons for high rating included experience in managing CVD patients, access to and familiarity with clinical practice guidelines and training in managing such patients, including PG training. Lack of training, unfamiliarity with guidelines, lack of self-confidence, fear of failure and lack of support from administrators and other health care providers were common reasons for a lower rating (≤7.0). When asked to rate their confidence in managing diabetes and hypertension on a scale of 1–10, 95.2% of physicians across six randomly selected villages of Bangalore Rural District, India, rated themselves 5 or above. Average confidence score was 6.21 ± 1.5 (George *et al*., 2016). The confidence level positively correlated with age and years of practice. A study aimed to evaluate providers’ perceived confidence in managing CVD risk factors in Botswana concluded that public sector health care providers in rural Botswana have low confidence in managing CVD risk factors (Gala *et al*., 2019). Most physicians outlined the aim of reaching the therapeutic range of blood pressure, blood glucose and lipids in their practice as one of the identifiers of effectiveness of treatment. Some outlined that they are able to gauge using the color-coding system consistent with WHO-PEN (Gupta *et al*., 2013). Some physicians subjectively assess treatment effectiveness through patients reporting on how well or how poorly their symptoms are controlled and whether the prescribed treatment has had an impact on the overall quality of life and functional status. The American Heart Association acknowledges that patient-reported health status is an important cardiovascular health outcome (Rumsfeld *et al*., 2013).

Participants were asked if our health system was keeping up with a more advanced or a developed health setting where the resources are more readily available. There was consensus among the participants that our response to CVD risk management is far from ideal. Factors that contribute to this perception include high doctor-patient ratio, unavailability of proper material resources to effectively manage CVDs, variable practice approaches among doctors as opposed to a standardized approach, unrealistic patient expectations, patient non-compliance to management plans and socio-economic status of patients. A study among French primary care physicians’ beliefs about CVD risk factors and best practices for managing CVD showed physicians were generally happy with their country’s health care system and cite equity as the primary reason. The physicians believe that they are successful at managing CVD risk factors by emphasizing aspects of the doctor–patient relationship, including spending more time with patients and focusing on education (Kalantan *et al*., 1999; Cherry *et al*., 2012; Friedberg *et al*., 2014).

Primary care physicians play a vital role in primary care. The primary care physicians in the study see their role as main or central and believe they are the central coordinators when it comes to managing patients. Physicians believe that they work toward early diagnosis and early management, thus aiming to reduce the complications among patients. The role of general practitioners in multidisciplinary groups studied in the Netherlands for the care of elderly defined the role of general practitioners as having the aptitude ‘to see the bigger picture’ (Grol *et al*., 2018). The importance of multidisciplinary approach has also been highlighted in another study (Jennings and Astin, 2017).

Prevention of CVDs has been the primary aim in a PHC setting. The physicians demonstrate a deep role in communicating with their patients and dealing with their lifestyle behaviors. Literature suggests that factors such as lack of training in counseling and motivational interviewing, time limitations, lack of space, language barriers, poor patient compliance and poor knowledge of lifestyle hinder patient care and motivation toward behavioral change (Lutala & Muula, 2022). In addition, physicians need to put importance to shared decision-making when it comes to patient management. This not only strengthens patient-physician trust and relationship but is a road to effective behavior change and patients feel important in their own self (Bonner *et al*., 2015; Ju *et al*., 2018). A PHC physician’s knowledge, attitude, beliefs and self-efficacy are proposed in a Precede-Proceed model which could be utilized as a guide in prevention practice toward positive behavior change (Makrides *et al*., 1997).

Very few participants had the knowledge on the exact or approximate values in trends and the burden of disease. The participants only generalized the statement that CVDs are a major concern. While it was encouraging to note that all participants acknowledged the seriousness of threats posed by CVDs, it is concerning that they lacked information on trends of the diseases in their own country. Similarly, participants in a study on knowledge, attitude and practice of hypertension in Nepal demonstrated PHC physicians in the study areas had unsatisfactory knowledge and practice on hypertension including knowledge of its epidemiology (Doroodchi *et al*., 2008; Karbach *et al*., 2011).

Most participants in this study stated that they believed patient expectations include ability of doctors to correctly advise them on all risk factors, for doctors to listen and understand their issues, do blood tests and other investigations to confirm diagnosis, prescribe correct medications, make them symptom-free, have regular follow-up clinics and be available to assist them even outside of clinic. A qualitative study in the Netherlands concluded that general practitioners’ perceptions about patient expectations seem justified: patients appear to have high hopes for testing as a diagnostic tool. They expect diagnostic certainty without mistakes and a proof of good health (van Bokhoven *et al*., 2006; Jaworski *et al*., 2017; Ni *et al*., 2017).

### Limitations

This study had some limitations. Participants of this study may not reflect the entire physician population in the country. It would be valuable to obtain perceptions of all other disciplines who are involved as a multidisciplinary team to deal with CVDs and its consequences. There were challenges found during data collection due to the current global pandemic, COVID-19. Also, face-to-face interview would have been more suitable and comprehensive in conducting in-depth interviews. The perceptions of physicians in a rural setting may have differed due to limited resources and patient, physician and health setting factors. With the recordings, it is also highly likely the participants would have given ideal answers and restricted their actual views and opinions.

## Conclusion

It is important to understand the perspective of physicians in primary care setting as they provide the first-line management to the undifferentiated population with CVDs. This study aimed to explore PHC physicians’ perceptions on their roles and their perceptions on management approaches on CVD risk reduction and management. This study demonstrated the perceptions of physicians on their CVD management skills including education, experience and trainings, beliefs and attitudes of physicians, self-confidence and effectiveness in CVD risk reduction. Also, their roles and expectations were explored, including perceptions of effective treatment, perceptions of their roles and perceptions of patients’ expectations. Physicians generally view their role as central and imperative. They perceive to be essential and leading toward combating CVDs. Some recommendations for the primary care physicians include uplifting self-motivation, strengthening personal abilities encouraging peer-supports groups and engaging in continuous medical education. The recommendations for the health ministry include addition of the tools and guidelines in the medical school curriculum (in collaboration with the Fiji National University and the University of Fiji) and internship curriculum, involving all medical officers in the SOPD settings, encouraging training and workshops for physicians, encouraging peer-support groups for physicians and strengthening the current protocols in place. Also, there is a need to evaluate the usefulness and regulation of the Fiji-CRAM and the WHO-PEN guidelines in these settings. Furthermore, there is need to train medical personnel on CVD assessment and management in PHC, together with emphasis on counseling techniques and motivational interviewing. The recommendations for future researchers include conducting a follow-up study in few years’ time as well as focusing on the multidiscipline involved in CVD risk reduction and management, conducting studies in all settings and including private sectors perceptions as well. It would also be vital to understand behavior change as an independent subject of interest among physicians.
